# *SNORA69* is up-regulated in the lateral habenula of individuals with major depressive disorder

**DOI:** 10.1038/s41598-024-58278-2

**Published:** 2024-04-09

**Authors:** Rixing Lin, Haruka Mitsuhashi, Laura M. Fiori, Ryan Denniston, El Cherif Ibrahim, Catherine Belzung, Naguib Mechawar, Gustavo Turecki

**Affiliations:** 1 Department of Psychiatry, McGill Group for Suicide Studies, Douglas Mental Health University Institute, McGill University, Montreal, QC Canada; 2 CNRS, INT, Institute Neuroscience Timone, Aix-Marseille Université, Marseille, France; 3 Imaging Brain and Neuropsychiatry iBraiN U1253, INSERM, Université de Tours, Tours, France

**Keywords:** Molecular neuroscience, Neuroscience, Molecular biology, Non-coding RNAs

## Abstract

Major depressive disorder (MDD) is a complex and potentially debilitating illness whose etiology and pathology remains unclear. Non-coding RNAs have been implicated in MDD, where they display differential expression in the brain and the periphery. In this study, we quantified small nucleolar RNA (snoRNA) expression by small RNA sequencing in the lateral habenula (LHb) of individuals with MDD (n = 15) and psychiatrically-healthy controls (n = 15). We uncovered five snoRNAs that exhibited differential expression between MDD and controls (FDR < 0.01). Specifically, *SNORA69* showed increased expression in MDD and was technically validated via RT-qPCR. We further investigated the expression of *Snora69* in the LHb and peripheral blood of an unpredicted chronic mild stress (UCMS) mouse model of depression. *Snora69* was specifically up-regulated in mice that underwent the UCMS paradigm. *SNORA69* is known to guide pseudouridylation onto 5.8S and 18S rRNAs. We quantified the relative abundance of pseudouridines on 5.8S and 18S rRNA in human post-mortem LHb samples and found increased abundance of pseudouridines in the MDD group. Overall, our findings indicate the importance of brain snoRNAs in the pathology of MDD. Future studies characterizing *SNORA69’s* role in MDD pathology is warranted.

## Introduction

Major depressive disorder (MDD) is a prevalent illness and is the leading cause of disability and premature death worldwide^1–3^. Despite the prevalence and burden of MDD the underlying etiology and pathological processes that occur in the brain of depressed individuals are still not well characterized. The lateral habenula (LHb) is a key brain structure mediating the communication between the forebrain and midbrain/hindbrain, thus, regulating monoaminergic neurotransmission^4–6^. It is also involved in pathways related to reward processing, stress adaptation, sleep/circadian rhythm, and its dysfunction is linked to maladaptive processing of negative and positive valence; all of which are core behaviors dysregulated in MDD^4–6^. While many molecular factors have been associated with MDD, the primary focus of past molecular studies has been the investigation of gene expression and systems that regulate gene expression^7–9^. More recently, numerous studies have highlighted the role of various non-coding RNAs in MDD, or antidepressant response, including microRNAs, long non-coding RNAs and circular RNAs and more recently small nucleolar RNAs^10–13^.

The term snoRNAs was coined in the early 1980s; however, the first identification of snoRNAs can be traced back to the early 1960s^14–17^. RNA sequencing has allowed the identification and annotation of over 200 snoRNAs in humans; however, it is estimated that there are over 1000 unique snoRNAs^18,19^. SnoRNAs are essential, medium length (60–300 nucleotides long), non-coding RNAs present in all eukaryotes. Conserved sequence motifs (“boxes”) present on all snoRNAs further separate snoRNAs into two main distinct classes. Box C/D snoRNAs contain box C (5′-RUGAUGA-3′) and box D (5′-CUGA-3′) sequence motifs located near the 5′ and 3′ end respectively. Box H/ACA snoRNAs contain box H (5′-ANANNA-3′) and a 5′-ACA-3′ sequence present near the 3’ end^19,20^. Box C/D and box H/ACA snoRNAs recruit distinct sets of partner proteins, forming a ribonucleoprotein (snoRNP) complex^19,20^. Classically, snoRNAs function by guiding chemical modifications onto ribosomal RNA (rRNA) or small nuclear RNAs (snRNAs), with box C/D snoRNAs guiding 2′-oxygen-ribose methylation and box H/ACA snoRNAs guiding pseudouridylation^19,20^.

In this study we used small RNA-sequencing, in human post-mortem LHb, to perform an un-biased profile of snoRNAs that are differentially expressed between individuals with depression and psychiatrically healthy controls.

## Materials and methods

### Human samples

The human post-mortem LHb samples included in this study partially overlap with those included in a previous study from our group^10^. LHb samples were obtained, in collaboration with the Quebec Coroner’s Office, from the Douglas-Bell Canada Brain Bank (Douglas Mental Health University Institute, Montreal, Quebec, Canada). All individuals were of French-Canadian origin, a homogeneous population with a well–documented founder effect^21^. We analyzed a total of 30 LHb samples (15 individuals with MDD, and 15 psychiatrically healthy controls) (Table 1). One sample in the control group and seven samples in the MDD group had presence of psychotropic drugs at the time of death. Individuals with MDD all died by suicide (hanging, intoxication, or jumping) while control subjects died by natural causes or by accident. Groups were matched for age, sex, post-mortem interval (PMI), RNA integrity number (RIN), and pH (Table 1). Psychological autopsies were performed as described previously, based on DSM-IV criteria^22^. Written informed consent was obtained from next-of-kin. This study and the methods described were approved by the Douglas Hospital Research Centre institutional review board and performed in accordance with relevant guidelines and regulations.

**Table 1:** Human Lateral Habenula Cohort Demographics.

	Control	MDD	p-value
Sex	5F/10 M	5F/10 M	N/A
Age	55.33 ± 5.12	54.27 ± 3.80	0.87
pH	6.35 ± 0.08	6.49 ± 0.07	0.18
RIN^1^	5.41 ± 0.44	5.98 ± 0.25	0.27
PMI^2^	62.45 ± 7.95	55.67 ± 6.51	0.51

(mean ± S.E.M.).

^1^RIN: RNA integrity number, ^2^PMI: post-mortem interval (in hours).

### Small RNA sequencing and analysis

RNA was extracted from all brain samples using a combination of the miRNeasy Mini kit and the RNeasy MinElute Cleanup kit (Qiagen), with DNase treatment, and divided into small (< 200 nt) and large (> 200 nt) fractions^10^. RNA quality was assessed using the Agilent 2200 Tapestation. Small RNA libraries were prepared from the small RNA fraction, using the Illumina TruSeq Small RNA protocol following the manufacturer’s instructions. Samples were sequenced at the McGill University and Genome Quebec Innovation Centre (Montreal, Canada) using the Illumina HiSeq2000 with 50nt single-end reads^10^. Adapters and low-quality bases (Phred score < 15) were trimmed with cutadapt v2.10. The exceRpt pipeline v4.6.3 with the options STAR_alignEndsType = EndToEnd, STAR_outFilterMismatchNmax = 0, and STAR_outFilterMatchNmin = 16 was used to align trimmed reads to the genome (GRCh38) and quantify the snoRNAs into counts. Only snoRNAs with greater than or equal to 10 counts in 70% of the samples within either the control or MDD group were retained for later steps. The svaseq function from the sva v3.46.0 was used to identify surrogate variables representing known or unknown factors significantly influencing our dataset for inclusion in the model for differential expression. DESeq2 v1.38.1 was used to normalize the counts with the median of ratios method and to perform differential expression analysis on our dataset.

### Validation

RNA was reverse transcribed to cDNA using M-MLV reverse transcriptase (Gibco) following the manufacturer’s protocol with random hexamers. Real-time quantitative PCR (RT-qPCR) reactions were run in triplicates using the QuantStudio 6 Flex Real-Time PCR System and data were collected using the QuantStudio Real-Time PCR Software v1.1 (Applied Biosystems). To measure RNA expression, we used custom-designed probes (Supplementary Table 1) with PowerUp SYBR Green Master Mix (ThermoFisher). Expression levels were calculated using the absolute quantitation standard curve method, using *U6* as an endogenous control^10^. Student’s t test and Pearson’s correlation were performed in GraphPad Prism 9.

### Mouse samples

Habenula samples were collected from unpredictable chronic mild stress (UCMS) and control male BALB/c mice from Hervé et al.,^23^. Mice underwent the UCMS exposure (UCMS; n = 8) or kept in standard housing conditions (control; n = 7) for eight weeks. Control mice were housed in groups of four in standard cages (21 × 38 cm) while UCMS mice were isolated in individual home cages (8.5 × 22 cm) with no physical contact with other mice. Stressors consisted of housing on damp bedding (~ 200 mL H_2_O for 100 g bedding), placement in an empty cage (home cage without bedding), placement in an empty cage with H_2_O (home cage without bedding filled with 1 cm H_2_O at 21 °C), switching cages, cage tilting (45°), predator sounds, introduction of predator feces/fur, inversion of light/dark cycle, and confinement in small tubes (4 cm diameter × 5 cm length). Stressors were varied and applied in different sequences per week to avoid habituation. Weight and coat state were measured weekly, as markers of UCMS-induced state of physiological stress, except for the last week before sacrifice. At the end of the eighth week, a complementary test of nest building, a common measure of well-being in mice, was performed just before sacrifice^24,25^. The nest building test is administered at the beginning of the activity period of the mice. The test mice are isolated in their home cages and pieces of pressed cotton are introduced in the cage. The quality of the nest is scored 5 h, and 24 h later according to the scale proposed by Deacon 2016^26^. Mice were euthanized by CO_2_ inhalation. For more detailed description please see Hervé et al.,^23^. All experiments on mice were carried out according to policies on the care and use of laboratory animals of European Community legislation 2010/63/EU and ARRIVE guidelines. The local Ethics Committee (Comité d’Ethique en Expérimentation Animale de Val de Loire (CEEAVdL) number 19) approved the protocols used in this study (protocol number 2011-06-10).

### *Snora69* expression

Total RNA was extracted from blood using Mouse RiboPure-Blood RNA isolation kit (Life Technologies) and from habenula using mirVana miRNA isolation kit (Life Technologies) according to manufacturer’s instructions. All RNA samples were subjected to DNase treatment. Concentration was assessed using nanodrop ND-1000 and RNA quality was assessed using Agilent 2100 Bioanalyzer. RNA was reverse-transcribed using M-MLV Reverse Transcriptase (200 U/µL) (ThermoFisher) with random hexamers. Expression of *Snora69* was assessed via RT-qPCR, as detailed above, using custom probes (Supplementary Table 1). Expression levels were calculated using the absolute quantitation standard curve method, using *U6* as an endogenous control. Student’s t test and Pearson’s correlation were performed in GraphPad Prism 9.

### Human neuronal culture

To test if antidepressant drugs have any effects on the expression of *SNORA69* we utilized human NPC cultures were as described in Fiori et al.^27^. Human neuronal cultures were screened for cytotoxic effects using the MTT assay, and antidepressants were applied at nontoxic concentrations as described by Lopez et al.,^28^. Following 2 weeks of neuronal differentiation, culture media was supplemented with duloxetine (10 µM; Sigma-Aldrich), escitalopram (100 µM; Sigma-Aldrich), haloperidol (10 µM; Sigma-Aldrich), lithium (1 mM; Sigma-Aldrich), Aspirin (1 mM; Sigma-Aldrich), or left untreated (control). Cells for each drug treatment were incubated for 48 h before being harvested for RNA extractions. Each drug treatment was performed in triplicate. RNA was extracted using the Zymo DirectZol RNA Extraction kit and cDNA construction and RT-qPCR were as described above. One-way ANOVA was performed in IBM SPSS Statistics version 27.

### N-cyclohexyl-N′-β-(4-methylmorpholinium) ethylcarbodiimide (CMC) treatment

The CMC treatment protocol was adapted from Antonicka et al. and Nagasawa et al., with slight modifications, to measure pseudouridylation levels in association with *SNORA69* expression^29,30^. CMC (Sigma-Aldrich) treatment was conducted on the above-mentioned RNA extracted from human post-mortem LHb samples. 1.5 ug of total RNA was used per sample and split into two separate aliquots (0.9 ug for CMC treatment and 0.6ug for negative control; each fraction was in a final volume of 20 uL). 2.9 uL of 40 mM EDTA (pH 8.0; Sigma-Aldrich) was pipette mixed, briefly centrifuged, placed into an 80 °C water bath for 3 min, and immediately placed on ice. Freshly made 0.4 M CMC was prepared in BEU buffer (50 mM bicine (pH 8.5; Sigma-Aldrich), 4 mM EDTA, and 7 M urea (Sigma-Aldrich)). 100 uL of CMC in BEU buffer or BEU buffer without CMC was added to each corresponding aliquot per sample (total of 20 RNA samples) pipette mixed and briefly centrifuged. Samples were incubated at 40 °C for 60 min at 1000 rpm with shaking. RNA was precipitated using 1000 uL 100% ethanol, 50 uL of 3 M sodium acetate (Sigma-Aldrich), and 1 uL glycogen (Invitrogen). Each sample was pipette mixed and briefly centrifuged before placing in − 80 °C for 30 min. Afterwards, samples were centrifuged at 16,000 g for 30 min at 4 °C. The supernatant was carefully discarded, 500 uL of cold 70% ethanol was added, and samples were centrifuged at 16,000 g for 10 min at 4 °C (this step was performed twice). After discarding the supernatant from the last ethanol wash, the samples were left to air dry. 30 uL of 50 mM sodium carbonate (pH 10.4; BioShop) and 2 mM EDTA was added to each sample, pipette mixed, briefly centrifuged, and incubated at 50 °C for 2 h at 1000 rpm with shaking. RNA was precipitated using 90 uL 100% ethanol, 3.5 uL of 3 M sodium acetate, and 1 uL glycogen; briefly centrifuged and placed in − 80 °C for 30 min and centrifuged at 16,000 g for 30 min at 4 °C. The supernatant was removed and 500uL of 70% ethanol was added and centrifuged at 16,000 g for 10 min at 4 °C (this step was performed twice). After discarding the supernatant from the last ethanol wash, the samples were left to air dry and then resuspended in 15 uL H2O.

### Pseudouridine (Ψ) quantification

RNA was reverse-transcribed as described above. We specifically quantified pseudouridylation at U^36^ on 18S rRNA and U^69^ on 5.8S rRNA using custom designed primers up- and down-stream of each pseudouridylation site (supplementary Fig. 1 & Supplementary Table 1). CMC treatment causes a stall in reverse transcription at pseudouridylated (Ψ) sites. This will cause a truncated fragment in CMC treated RNA compared to non-CMC treated RNA (Supplementary Fig. 1). The abundance of Ψ is quantified by the ratio (R) of fold-change of non-truncated fragments (long fragment) with or without CMC treatment to the fold-change of truncated fragments (short fragment) with or without CMC treatment using the following equation:

$$R=\frac{{2}_{long\, fragment}^{\Delta\, Ct\, long\, fragment (nonCMC\, mean-CMC \,mean)}}{{2}_{short\, fragment}^{\Delta\, Ct\, short\, fragment (nonCMC\, mean-CMC\, mean)}}$$

To better visualize the ratio, we plotted the inverse ratio (1/R) labeled as “relative Ψ”. Mann–Whitney U test and Pearson’s correlation was performed in GraphPad Prism 9.

## Results

### *SNORA69* is up-regulated in the lateral habenula of depressed humans and mice

**Figure 1:**
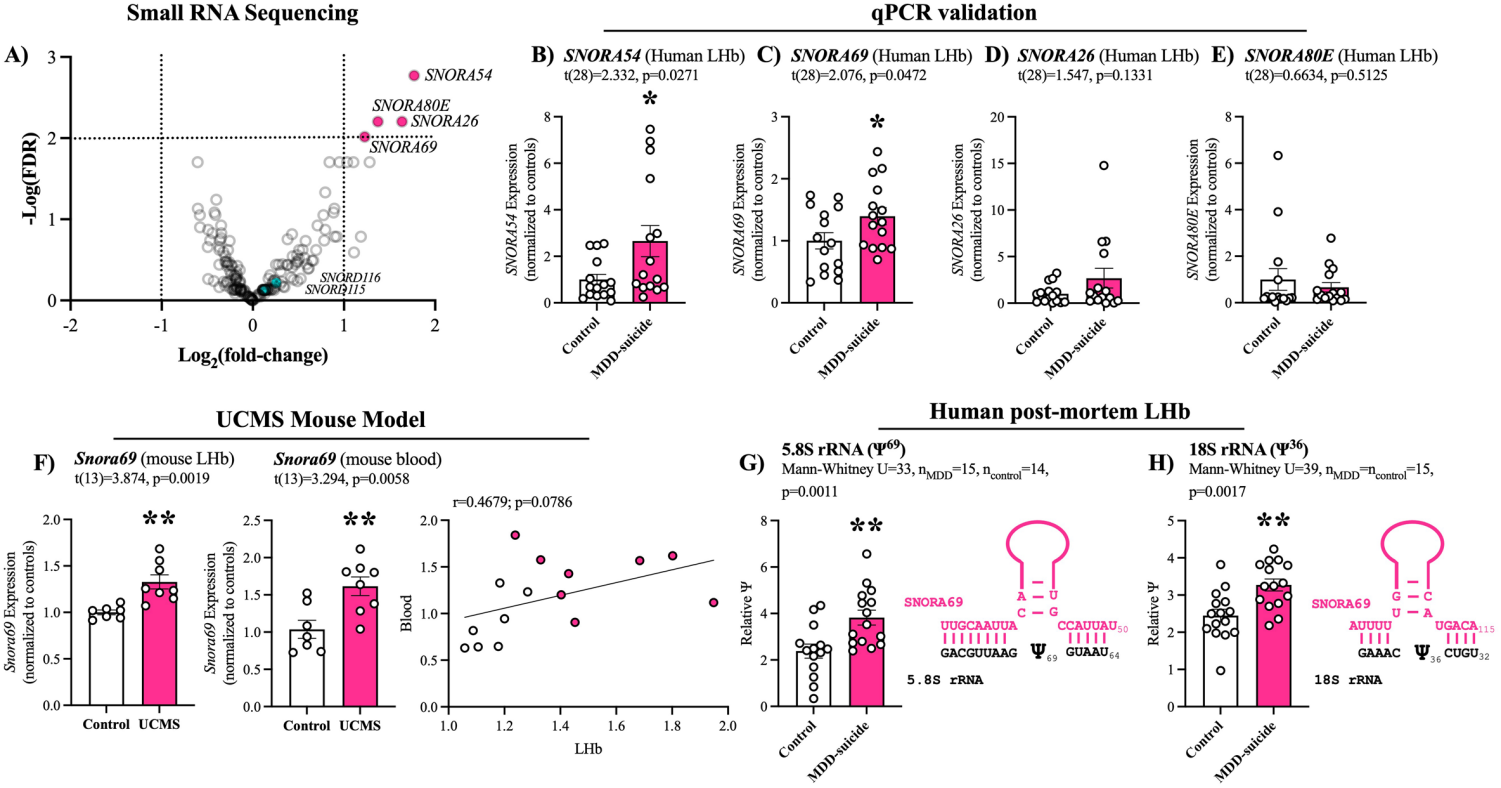
*SNORA69* is up-regulated in the LHb of individuals with MDD and regulates pseudouridine abundance. (**A**) Volcano plot of 184 snoRNA detected from small RNA sequencing in post-mortem human LHb tissue. We applied a cut-off of FDR < 0.01 resulting in four candidate snoRNAs: *SNORA54*, *SNORA80E*, *SNORA26*, and *SNORA69*. We also highlight *SNORD115* and S*NORD116* although they did not reach significance. (**B-E**) RT-qPCR validation of candidate snoRNAs. Only SNORA54 (**B**) and SNORA69 (**C**) were significantly validated. (**F**) *Snora69* expression was significantly up-regulated in the LHb (left) and pheripheral blood (center) of mice that underwent unpredicted chronic mild stress (UCMS). *Snora69* expression was positively correlated between the LHb and peripheral blood (right). White circles are controls and pink circles are UCMS. (**G**, **H**) *SNORA69* guides pseudouridylation onto 5.8S rRNA (**G**) and 18S rRNA (**H**). We observed an increased detection of pseudouridines on 5.8S rRNA (**G**) and 18S rRNA (**H**) in individuals with MDD compared to controls. All bar plots represent the mean with individual data points as dots. Ψ denotes pseudouridylation event and the superscript number indicates the nucleotide position on the *SNORA69* sequence. Error bars represent S.E.M. Student’s two-tailed t test (**B-F**) or Mann–Whitney U test (**G-H**) were used to assess group differences. Pearson correlation coefficient (r) was used for correlation analysis. *p < 0.05, **p < 0.01.

We performed small RNA sequencing in 30 samples (15 individuals with MDD, and 15 psychiatrically healthy controls) from the lateral habenula (LHb). Groups were matched for age, sex, post-mortem interval (PMI), pH, and RIN (Table 1). We identified 184 unique snoRNAs that passed filtering (as described in the methods section), with 11 snoRNAs displaying significant (FDR < 0.05) differential expression between MDD and controls (Supplementary Table 2). To narrow down snoRNA candidates for technical validation we applied a more stringent FDR < 0.01 which left use with *SNORA54*, *SNORA80E*, *SNORA26*, and *SNORA69* (Fig. 1A & Supplementary Table 2). Of the four candidate snoRNAs, *SNORA69* and *SNORA54* passed RT-qPCR validated (Fig. 1B–E).

Previous studies suggest that the LHb acts as a relay center for forebrain limbic regions and midbrain monoaminergic centers; playing a central role in reward or aversion related learning and memory associated to stressful and aversive stimuli^31–33^. Thus, we investigated the expression of our candidate snoRNAs in an unpredictable chronic mild stress (UCMS) mice model, a common model of depression^23,34,35^. Given that *SNORD54* (Accession No. XR_004934678.1) does not have a well-defined ortholog in mice, we focused our attention exclusively on *SNORA69* (Accession No. NR_002900.1) moving forward^36^. UCMS mice showed a significant increase in weight gain compared to unstressed mice by the end of the UCMS protocol. Additionally, depressive-like physical alterations, evaluated through coat state score, distinguished unstressed from UCMS mice, while assessment via the nest-building test revealed reduced motivation and apathetic behavior in UCMS mice^23^. Similar to what we observed in humans, *Snora69* was significantly elevated in UCMS mice, in both the LHb and peripheral blood samples (Fig. 1F). *Snora69* also displayed positive correlation between the LHb and peripheral blood suggesting *Snora69* may be a potential biomarker candidate for MDD, although this did not reach statistical significance (Fig. 1F).

### *SNORA69* expression is not altered by antidepressant drugs

We further investigated if antidepressant drugs have effects on *SNORA69* expression by using human neuronal cultures treated with four different psychotherapeutic drugs (duloxetine, escitalopram, haloperidol, and lithium), a non-psychotropic drug control (aspirin) and no drug control. Interestingly, the expression of *SNORA69* was not significantly altered by any of the abovementioned drugs (Supplementary Fig. 2).

### *SNORA69* is associated with an increase in pseudouridylation on 5.8S rRNA and 18S rRNA

Pseudouridine is an abundant RNA modification and affects the structure of the modified RNA and their interactions with protein and/or RNA binding partners, making this modification especially important in rRNA functioning^37^. *SNORA69* is known to guide pseudouridylation onto 5.8S rRNA U^69^ and 18S rRNA U^36^ (Fig. 1G,H)^38–41^. We investigated the amount of pseudouridines at these two locations on each corresponding rRNA in our human LHb samples. We observed a significant increase of pseudouridines on both 5.8S rRNA (U^69^) and 18S rRNA (U^36^) (Fig. 1G,H). However, correlation between *SNORA69* expression and pseudouridylation was only observed for 18S rRNA (Supplementary Fig. 3A). There were no significant differences in the expression of either 5.8S rRNA nor 18S rRNA and no significant correlation between the expression of *SNORA69* and expression of each corresponding rRNA (Supplementary Fig. 3B-C). This indicates that *SNORA69* is not influencing the quantities of 5.8S rRNA and 18S rRNA, but more likely influencing the functioning of these rRNA through increasing the amount of pseudouridylation.

## Discussion

Despite the rapidly growing body of work highlighting the role of non-coding RNAs in MDD, as well as other psychiatric conditions, the involvement of snoRNAs in psychiatric disorders remains under investigated^11–13,28,42^. Our study suggests that snoRNA expression, in the LHb, are associated with MDD. From our discovery analysis we identified 184 unique snoRNAs with 22% falling into the H/ACA box snoRNA family and 74% falling into the C/D box snoRNA family. Of the snoRNAs identified, 11 snoRNAs were differentially expressed in individuals with MDD and died by suicide. It’s interesting to note that although that majority of snoRNAs identified were C/D box snoRNAs those that were significantly differentially expressed in MDD were primarily H/ACA box snoRNAs. Of the top 11 snoRNAs seven were H/ACA box snoRNAs whereas only three were C/D box snoRNAs. Moreover, the top four snoRNAs that passed a more stringent FDR < 0.01 cutoff were all H/ACA box snoRNAs. The exact meaning and implication of these observations is unclear, but perhaps there might be some association between common mechanisms that elicit pseudouridylation onto target rRNAs with those involved in MDD. Upon technical validation and investigation in a UCMS mouse model, our study points to *SNORA69* to be strongly associated with MDD. Furthermore, in our cell culture experiments, *SNORA69* expression was not altered by psychotropic drugs including antidepressants.

Previous studies suggest that the dorsal raphe and LHb have reciprocal projections and that decreased serotonin transmission can disinhibit the LHb possibly mediating depressive symptoms^43–45^. Reciprocally the LHb has also been shown to modulate dorsal raphe nuclei activity^44,45^. This of particular interest since our observations for *SNORA69* seem to be specific to MDD without any expressional change when exposed to antidepressant treatment, which primarily targets the serotonergic system.

While no previous studies have directly investigated snoRNAs in the context of MDD several studies have demonstrated the importance of snoRNAs in the brain^46,47^. Specifically, a cluster of brain enriched snoRNAs including *SNORD115* (*H/MBII-52*) and *SNORD116* (*H/MBII-85*) have received attention for their potential involvement in Prader-Willi syndrome (PWS). PWS is a complex genetic disorder caused by the loss of function of paternally imprinted genes in the 15q11-q13 region, of which *SNORD115* and *SNORD116* clusters are located^48,49^. Several studies have created mice models where *Snord116* is knocked out (KO) and mice exhibit phenotypic behaviors reminiscent of PWS^50–54^. Although it remains unclear how *SNORD116* contributes to PWS, studies have shown that *SNORD116* can influence gene expression and DNA methylation^55,56^. *SNORD115* has been shown to regulate the editing or alternative splicing of serotonin receptor 2C^57–59^. *SNORD115* KO studies in mice do not result in robust changes in behavior; however, *SNORD115*-KO mice present changes in monoaminergic circuits specifically increasing the firing rate of dopaminergic neurons in the ventral tegmental area and serotonergic neurons in the dorsal raphe nucleus^60,61^. We note that, although we did identify *SNORD116* and *SNORD115* in our human post-mortem LHb samples, neither were significantly differentially expressed between MDD and controls (Fig. 1A).

Using small RNA sequencing we were able to associate *SNORA69* with MDD. However, we recognize that our study is not without limitations. The sample size of our human and mouse LHb cohort is limited, but robust enough to identify and validate *SNORA69*. Additionally, our study focused on snoRNA expression in the LHb, precluding investigation in other brain areas implicated in MDD. While we did not observe any effect of *SNORA69* expression with antidepressant treatment, our investigation was confined to human neuronal culture whereas different cell types may have different outcomes. Although we demonstrated that an up-regulation of *SNORA69* is associated with an increase of pseudouridylation onto 5.8S and 18S rRNA, the direct relevance of this observation to the psychopathology of MDD remains uncertain. We speculate that alterations to rRNA pseudouridylation will influence binding to tRNA and mRNA, ultimately having implications on translation and proteomic profiles in MDD; however further studies are needed to confirm this^62,63^. Moreover, while the UCMS mouse model is recognized as a well-established mouse model of depression, we acknowledge the limitation of not conducting a comprehensive battery of behavioral tests on these animals. Lastly, due to the heterogenous nature of MDD and of the subjects included in the study, it remains unclear if *SNORA69* has a general association with MDD or with a confounding factor that may be commonly associated with MDD. Further work is needed to better characterize the role of *SNORA69* in MDD. Furthermore, our mouse data suggest that the expression of *Snora69* is positively correlated between LHb and peripheral blood making it an interesting candidate for further investigations as a predictive biomarker for MDD or mediator marker contributing to MDD pathology.

### Supplementary Information

Supplementary Information 1.

Supplementary Table 2.

## Data Availability

Small RNA sequencing data used in this article can be obtained from NCBI GEO under accession number GSE244651.
