# Supplementary material for: SNORA69 is up-regulated in the lateral habenula of individuals with major depressive disorder
Source: Sci Rep. 2024 Apr 9;14:8258. doi: 10.1038/s41598-024-58278-2 (PMC11001866; doi:10.1038/s41598-024-58278-2)
Supplement: Supplementary file 1 — Supplementary Information 1. [file 41598_2024_58278_MOESM1_ESM.docx]

SUPPLEMENTARY MATERIALS FOR

**SNORA69 is up-regulated in the lateral habenula of individuals with major depressive disorder**

Rixing Lin, Haruka Mitsuhashi, Laura M. Fiori, Ryan Denniston, El Cherif Ibrahim, Catherine Belzung, Naguib Mechawar, and Gustavo Turecki^*^

*Corresponding author. Email: [gustavo.turecki@mcgill.ca](mailto:gustavo.turecki@mcgill.ca)

**This document includes:**

Supplementary Figure 1

Supplementary Figure 2

Supplementary Figure 3

**Other supplementary materials for this manuscript includes the following:**

Supplementary Table 1

Supplementary Table 2 (external excel file)

**
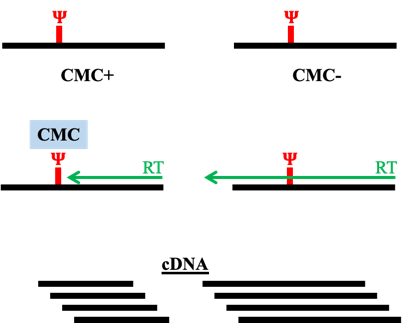
Supplementary Figure 1.** Schematic diagram of pseudouridine detection (*related to Materials and Methods and Figure 1F*).

Schematic diagram depicting how N-cyclohexyl-N’-β-(4-methylmorpholinium) ethylcarbodiimide (CMC) treatment is used to quantify pseudouridylation. CMC treatment causes a stall in reverse transcription at pseudouridylated (Ψ) sites. This will cause a truncated fragment in CMC treated RNA compared to non-CMC treated RNA. The abundance of Ψ is quantified by the ratio (R) of fold-change of non-truncated fragments (long fragment) with or without CMC treatment to the fold-change of truncated fragments (short fragment) with or without CMC treatment.

**Supplementary Figure 2**. Psychotropic drugs do not alter SNORA69 expression in human NPC culture

Expression of *SNORA69* in human neuronal cultures treated with various antidepressants (duloxetine and escitalopram), other psychotropic drugs (haloperidol and lithium), control drug (aspirin), and regular culturing media (control). One-way ANOVA; bar plots represent the mean with individual data points as dots. Error bars represent S.E.M.

**
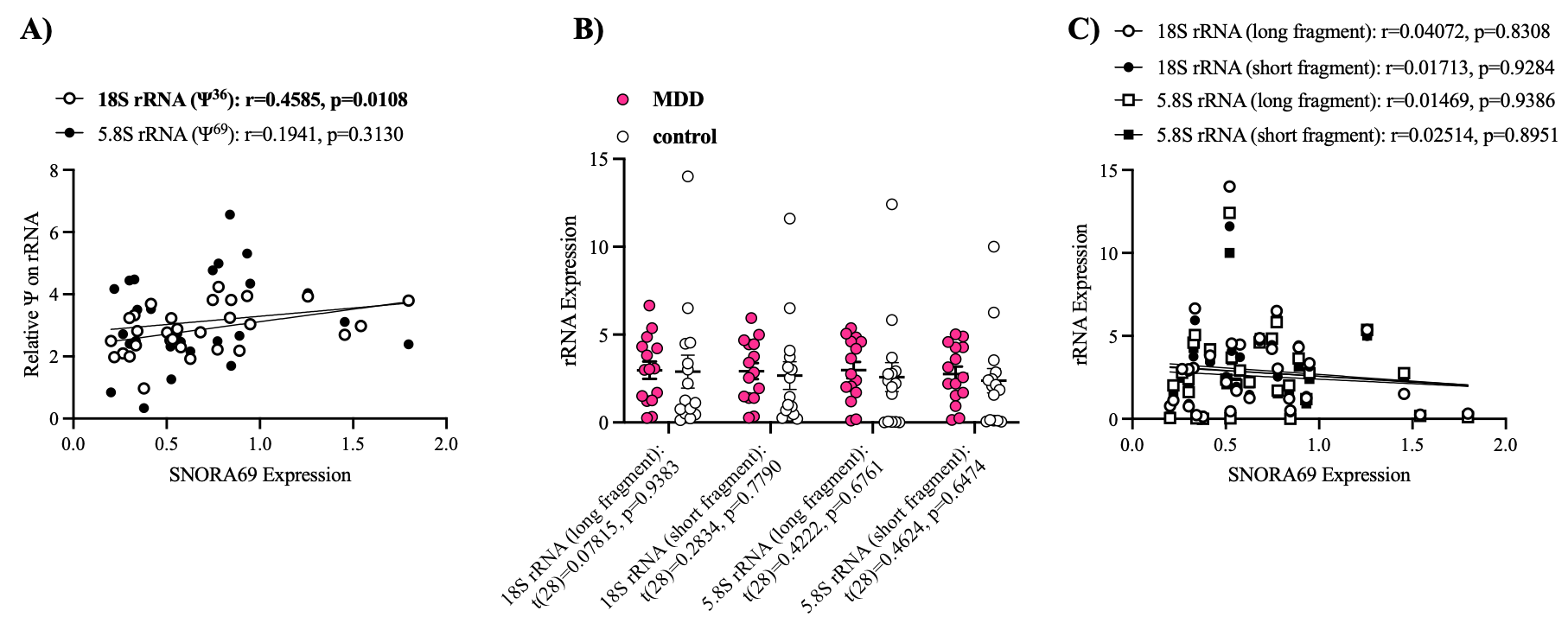
**

**Supplementary Figure 3.** Pseudouridylation on 5.8S and 18S rRNA (*related to Figure 1G-H*)

**A)** Correlation between pseudouridylation on 5.8S/18S rRNA and SNORA69 expression. **B)** Expression of 5.8S and 18S rRNA between MDD (pink) and controls (white). Short and long fragments refer to primer pairs used in pseudouridylation quantification. **C)** Correlation between 5.8S/18S rRNA expression and SNORA69 expression. Student’s two-tailed t tests were used to assess group differences. Pearson correlation coefficient (r) was used for correlation analysis. All bar plots represent the mean with individual data points as dots. Error bars represent S.E.M.

| Target | Sequence |
| --- | --- |
| homo-SNORA54 forward | GAGCACTGTTCGTAACCCGT |
| homo-SNORA54 reverse | GTCAGTCATGTGTCGCTGGA |
| homo-SNORA80E forward | TCTGTGGGCCTCTCATAGTGTA |
| homo-SNORA80E reverse | TGCTCACAGCCCACAGGTAA |
| homo-SNORA26 forward | GGTTGACCCAGTGCTTTAAGA |
| homo-SNORA26 reverse | AGCTTTCCAGTCTCTTCTCTGG |
| homo-SNORA69 forward | CAGGTTGCAATTACAGTGCTTCA |
| homo-SNORA69 reverse | TAACACGGCTTTTCTTTCAGCA |
| mus-Snora69 forward | GCAATTACAGTGCTTCATTTTGTGG |
| mus-Snora69 reverse | CTGTTCATGTCAAGGTGTCACG |
| homo/mus-U6 forward | CGCTTCGGCAGCACATATAC |
| homo/mus-U6 reverse | TGGAACGCTTCACGAATTTGC |
| 5.8S short fragment forward | GCAGGACACATTGATCATCG |
| 5.8S long fragment forward | GCGAGAATTAATGTGAATTGC |
| 5.8S universal reverse | GACGCTCAGACAGGCGTAGC |
| 18S short fragment forward | ATTAAGCCATGCATGTCTAAG |
| 18S long fragment forward | TGCTTGTCTCAAAGATTAAGC |
| 18S universal reverse | GTTATCCAAGTGGGAGAGGAG |

**Supplementary Table 1.** RT-qPCR Primers
